# TRAJECTORIES OF FALLS AND ASSOCIATED FACTORS AMONG ADULTS 65 AND OLDER: INSIGHTS FROM THE HEALTH AND RETIREMENT STUDY

**DOI:** 10.1093/geroni/igae098.4389

**Published:** 2024-12-31

**Authors:** Rohit Baal Balasundaram, Brittany Krenek-Schneider, Edna Patricia Mendoza, Anup Dharmendrakumar Patel, Lien Quach, Uyen-Sa Nguyen

**Affiliations:** University of North Texas Health Science Center, Fort Worth, Texas, United States; University of North Texas Health Science Center, Fort Worth, Texas, United States; University of North Texas Health Science Center, Fort Worth, Texas, United States; University of North Texas Health Science Center, Fort Worth, Texas, United States; Massachusetts General Hospital, Boston, Massachusetts, United States; University of North Texas Health Science Center, Fort Worth, Texas, United States

## Abstract

Falls are the leading cause of injuries among older adults in the United States, with over 14 million older adults, or 1 in 4, reporting a fall each year. This longitudinal prospective cohort study investigated fall trajectories and associated risk factors in people with no prior history of falls. We analyzed 2006 to 2018 data from the Health and Retirement Study (HRS), restricted to participants 65+ who did not report falls prior to the baseline year of 2006. The outcome is biennial incidence of falls, and risk factors include sociodemographic and biological factors, and health behaviors. Group-based trajectory modeling identified distinct fall trajectories. Multinomial logistic regression assessed associations between trajectories and risk factors;. Three distinct trajectories of falls incidence were identified over a 12-year period. Nearly half of the population (48.9%) remained in a low-stable risk group, 22.4% experienced a moderate but steadily increasing risk, and 28.7% belonged to a high-increasing risk group. Individuals identified as Black/African American or “Other” race were associated with a lower likelihood of being in the high-risk group compared to White/Caucasian individuals (OR = 0.38, 95% CI: 0.27, 0.55; OR = 0.32, 95% CI: 0.16, 0.67, respectively). Conversely, having comorbid conditions was associated with a higher likelihood of being in the high-risk group (OR = 1.37, 95% CI: 1.08, 1.73). The study identified distinct trajectories of fall risk among older adults without a history of falls, significantly varying by race/ethnicity and comorbid conditions, highlighting the need for targeted fall prevention strategies tailored to specific groups.

